# Neurological Complications in Intensive Care Units: From Delirium to Long-Term Cognitive Dysfunction—A Narrative Review

**DOI:** 10.3390/jcm15072478

**Published:** 2026-03-24

**Authors:** Mateusz Szczupak, Jacek Kobak, Jolanta Wierzchowska, Amelia Dąbrowska, Wioletta Mędrzycka-Dąbrowska, Sabina Krupa-Nurcek

**Affiliations:** 1 Department of Anaesthesiology and Intensive Therapy in the Nicolaus Copernicus Hospital in Gdańsk, 80-808 Gdansk, Poland; szczupak.mateusz@icloud.com (M.S.); jwierzchowska@copernicus.gda.pl (J.W.); 2 Department of Otolaryngology, Faculty of Medicine, Medical University of Gdańsk, 80-210 Gdansk, Poland; 3 Department of Otolaryngology, University Clinical Center, 80-214 Gdansk, Poland; 4 Faculty of Medicine, Lazarski University in Warsaw, 02-662 Warsaw, Poland; amelia92012@gmail.com; 5 Department of Anaesthesiology Nursing and Intensive Care, Faculty of Health Sciences, Medical University of Gdańsk, 80-211 Gdansk, Poland; wioletta.medrzycka-dabrowska@gumed.edu.pl; 6 Department of Surgery, Faculty of Medicine, Collegium Medicum, University of Rzeszów, 35-959 Rzeszow, Poland; sabinakrupa@o2.pl

**Keywords:** intensive care unit, delirium, acute brain dysfunction, encephalopathy, seizures, PICS

## Abstract

**Background/Objective:** Advances in intensive care medicine have substantially improved the survival of critically ill patients; however, they have also revealed the growing burden of neurological complications that affect both short-term outcomes and long-term functioning. Neurological complications in the intensive care unit (ICU) include a wide spectrum of disorders, ranging from acute brain dysfunction such as delirium, coma, and encephalopathy to persistent cognitive impairment after discharge, which represents a key component of Post-Intensive Care Syndrome (PICS). Delirium affects approximately one-third of ICU patients and is independently associated with increased mortality, prolonged hospitalization, and worse long-term neurocognitive outcomes. Due to the limited effectiveness of pharmacological therapies, current clinical approaches emphasize prevention, early diagnosis, and non-pharmacological strategies in line with PADIS guidelines. This narrative review aims to provide a clinically relevant synthesis of neurological complications in adult ICU patients, conceptualized as a continuum from acute brain dysfunction to long-term cognitive impairment. **Methods:** A narrative review of the literature was conducted, focusing on studies addressing epidemiology, pathophysiology, risk factors, diagnostic strategies, and prevention of neurological complications in critically ill adults. Attention was given to delirium, ICU-acquired cognitive impairment, and their association with PICS, as well as to current guideline-based and non-pharmacological interventions. **Results:** Available evidence indicates that neurological complications in the ICU are multifactorial and result from the interaction between patient vulnerability, severity of illness, systemic inflammation, sedative exposure, and environmental factors. Delirium remains the most common manifestation of acute brain dysfunction and is strongly associated with adverse outcomes. Increasing evidence supports the effectiveness of structured screening, early mobilization, sleep optimization, and multidisciplinary care bundles in reducing delirium incidence and duration. Moreover, growing attention is directed toward post-ICU follow-up and rehabilitation to reduce long-term cognitive decline. **Conclusions:** Neurological complications should be considered a central component of critical illness and a continuum extending beyond ICU discharge. Early identification of high-risk patients, implementation of preventive strategies, and integration of acute and post-ICU care are essential to improve survival and long-term cognitive outcomes. Further research should focus on personalized preventive and neuroprotective approaches in critically ill patients.

## 1. Introduction

Progress in intensive care over recent decades has led to a significant reduction in mortality rates for critically ill patients, while also revealing the growing importance of long-term complications following hospitalization in the intensive care unit (ICU). Neurological complications are particularly important, impacting not only the acute phase of the illness but also quality of life, cognitive functioning, and independence after discharge [1,2,3].

ICU delirium is currently recognized as a clinical manifestation of acute brain dysfunction and is one of the most common neurological complications in critically ill patients. Meta-analyses indicate that it occurs in approximately 30% of ICU patients, with the hypoactive form predominating, often underdiagnosed in clinical practice [4]. The presence of delirium is associated with increased mortality, prolonged duration of mechanical ventilation and ICU stay, and poorer functional outcomes after discharge [5].

In response to the scale of the problem, guidelines for ICU management have been developed, including the 2018 SCCM PADIS recommendations and their 2025 update, which emphasize the importance of systematic delirium screening, optimization of analgesic sedation, and the implementation of non-pharmacological interventions [1,2]. At the same time, large randomized clinical trials have demonstrated limited efficacy of pharmacotherapy in shortening delirium duration, leading to a shift in management toward preventive and multi-component strategies [3,6].

Neurological complications of the ICU, however, are not limited to delirium. Critical illness encephalopathies, particularly septic encephalopathy, seizures, cerebrovascular complications, and ICU-acquired weakness, significantly complicate treatment and impact prognosis. Increasing attention is also being paid to long-term cognitive impairment after the ICU, a key component of Post-Intensive Care Syndrome (PICS) [7,8,9].

## 2. Aim of Study

The aim of this narrative review is to synthesize the current evidence regarding neurological complications in adult patients treated in intensive care units, with a focus on the clinical continuum from acute brain dysfunction in the ICU to long-term cognitive sequelae after discharge. Particular emphasis is placed on the role of delirium as a central phenotype of acute brain dysfunction, associated with both short- and long-term outcomes [5].

## 3. Materials and Methods

This article is a narrative review and was designed to synthesize the current state of knowledge regarding neurological complications in adult patients treated in intensive care units.

A literature search was conducted in the following databases: PubMed, Web of Science, EBSCO, the Cochrane Library, and Google Scholar. The search included publications published between 1 January 2015, and 5 November 2025, available in full text or as authoritative online bibliographic records. Combinations of keywords and MeSH terms were used, including intensive care unit, critical illness, delirium, acute brain dysfunction, encephalopathy, sepsis-associated encephalopathy, long-term cognitive impairment, post-intensive care syndrome, and PICS. The search strategy was adapted to the specific needs of each database.

### 3.1. Inclusion and Exclusion Criteria

The review included:

- Clinical guidelines,
- Systematic reviews and meta-analyses,
- Randomized clinical trials,
- Large observational studies (cohort, multicenter), involving adult ICU patients and addressing acute or long-term neurological complications of intensive care.

The following papers were excluded:

- Relating to the pediatric population,
- Focusing exclusively on primary neurointensivist diseases (e.g., isolated brain injuries, intracranial hemorrhages without the context of critical illness),
- Published before 2015,
- Unavailable in full text or of low methodological value,
- Papers written in a language other than English.

### 3.2. Data Synthesis

Identified publications were qualitatively analyzed, with particular emphasis on the consistency of findings, clinical significance, and currency of the data. Results were presented in a narrative synthesis, grouping the evidence into key thematic areas: delirium in the ICU, critical illness encephalopathies, and long-term cognitive impairment after ICU discharge. Priority was given to publications with the highest level of methodological validity.

The methodology was selected to provide a comprehensive, practical approach to the problem that addresses the clinical needs of anesthesiologists and intensivists.

## 4. Definitions and Classifications of Neurological Complications in the ICU

Neurological complications in adult patients treated in the intensive care unit constitute a heterogeneous group of disorders involving the central nervous system (CNS), peripheral nervous system, and muscles. Their clinical presentation can be acute—hours/days—or late—weeks/months after discharge. In clinical practice, a useful tool for assessing neurological complications is a classification based on 1. localization (CNS vs. peripheral nervous system/muscles), 2. dynamics (acute vs. long-term), and 3. predominant clinical phenotype (impaired consciousness/attention, focal neurological deficits, seizures, generalized weakness) [1,10].

### 4.1. Acute Brain Dysfunction in the ICU: Delirium, Coma, and Encephalopathy

The PADIS guidelines recommend systematic assessment of pain, sedation depth, and delirium in adult ICU patients, considering these elements as fundamental components of the concept of “acute brain dysfunction” in intensive care [1,2]. Delirium is a syndrome with an acute onset and fluctuating course, defined by disturbances of attention and consciousness, and coexisting cognitive deficits. In the ICU environment, clinical diagnosis can be complicated by sedation, mechanical ventilation, and coexisting encephalopathies; therefore, routine screening using validated tools, primarily the CAM-ICU or ICDSC, is recommended [1,11]. Severity measures (e.g., CAM-ICU-7; ICDSC as a spectral scale) are also increasingly used in research and risk stratification, enabling a shift away from a binary approach and better linking clinical status with prognosis [11,12].

Coma refers to the inability to awaken or respond to stimuli and, in the ICU, most often results from the severity of the underlying disease or pharmacological sedation. Encephalopathy, on the other hand, is an umbrella term encompassing global brain dysfunction of metabolic, toxic, hypoxic, or inflammatory etiology; clinically, it can range from confusion to coma and often coexists with delirium, constituting a key element of the differential diagnosis [1,11].

### 4.2. Septic Encephalopathy as a Model of Critical Illness Encephalopathy

Among the encephalopathies observed in the ICU, sepsis-associated encephalopathy (SAE) is particularly important. It is defined as brain dysfunction secondary to sepsis in the absence of direct CNS infection, detectable structural changes, or other dominant cause of encephalopathy [7,13]. Current studies emphasize the lack of a single “gold standard” for diagnosis: SAE is a clinical diagnosis that requires excluding alternative causes of impaired consciousness and cognitive function. Pathophysiology includes neuroinflammation, blood–brain barrier dysfunction, and microcirculation disorders [7,14].

### 4.3. Seizure Activity and Nonconvulsive Status Epilepticus in Critically Ill Patients

In critically ill patients, seizures and non-convulsive status epilepticus (NCSE) may present with minimal symptoms, for example, as persistent disturbances of consciousness despite optimal sedation and correction of metabolic disturbances. Electroencephalography plays a key diagnostic role, and the ACNS Standardized Critical Care EEG Terminology (2021 version), widely used in practice and research, standardizes the description and interpretation of EEG patterns in the ICU [15,16].

### 4.4. Peripheral Complications: ICU-Acquired Weakness

ICU-acquired weakness (ICU-AW) is defined as clinically detectable, generalized, and usually symmetric muscle weakness that develops during the course of critical illness and is unexplained by another, more likely etiology. It primarily includes critical illness polyneuropathy (CIP) and critical illness myopathy (CIM), which significantly complicates withdrawal from mechanical ventilation and is associated with poorer functional outcome after discharge [10,17].

### 4.5. Long-Term Sequelae: The Cognitive Component of Post-Intensive Care Syndrome

Long-term neurological consequences of ICU treatment are currently conceptualized as Post-Intensive Care Syndrome (PICS), encompassing new or exacerbated deficits in the physical, mental, and cognitive domains following critical illness [9,18]. In the context of this manuscript, the cognitive domain of PICS is crucial, as it is associated with reduced independence and quality of life and should be considered as a potential “prolongation” of acute brain dysfunction after ICU discharge [5,9].

## 5. Epidemiology and Clinical Burden

Neurological complications in the ICU are common and significantly impact both short-term outcomes (mortality, duration of mechanical ventilation, length of hospitalization) and post-discharge functioning. This chapter focuses on three domains with the best-documented epidemiology and clinical consequences: delirium, ICU-acquired weakness, and long-term cognitive impairment after the ICU.

### 5.1. Incidence of Delirium in the ICU

A meta-analysis by Salluh et al., including 42 studies (16,595 patients assessed using validated tools), showed that delirium was diagnosed in 31.8% of critically ill patients [5]. A systematic review and meta-analysis by Krewulak et al. (48 studies, 27,342 patients) confirmed a prevalence of approximately 31% and a predominance of the hypoactive phenotype: 17% for hypoactive delirium, 10% for mixed delirium, and 4% for hyperactive delirium (with significant heterogeneity) [4]. More recent analyses indicate that the prevalence of delirium remains high in real-world studies, despite the implementation of preventive strategies based on the ABCDEF approach, underscoring the complexity of the pathophysiology and the limitations of single interventions [19,20,21,22].

### 5.2. Clinical Burden and Consequences of Delirium

Delirium in the ICU is not merely a transient disturbance of consciousness, but an event with measurable prognostic significance. In the meta-analysis by Salluh et al., delirium was associated with a significantly higher risk of death during hospitalization (RR 2.19; 95% CI 1.78–2.70), longer duration of mechanical ventilation, and prolonged ICU and hospital stay [5]. The authors also noted the association between delirium and cognitive decline after discharge, which constitutes a clinical bridge between acute brain dysfunction in the ICU and late neurological sequelae [5]. More recent observational studies confirm that delirium is not only a marker of disease severity but also an independent predictor of poorer long-term survival and limited functional recovery [23].

### 5.3. ICU-Acquired Weakness—Epidemiology and Consequences

ICU-acquired weakness is one of the most common neuromuscular complications of critical illness. In a systematic review by Appleton et al., the incidence of ICU-AW (CIP, CIM, or mixed form) was 40% (95% CI 38–42%) and was higher in studies using electrophysiological diagnostics than in analyses based solely on clinical assessment [24]. Recent reviews indicate that ICU-AW prolongs mechanical ventilation and increases the risk of extubation failure, leading to long-term functional limitations after discharge and posing a significant burden on patients, their caregivers, and the healthcare system [25,26].

### 5.4. Persistent Cognitive Dysfunction Following Intensive Care

Data indicate that cognitive impairment after ICU discharge is common and may persist for months or years. A meta-analysis by Ho et al. (58 studies, 347,940 patients) reported a prevalence of cognitive impairment of 45–50% in the first 6 months after discharge, with subsequent stabilization to approximately 30% after 12 months of follow-up [27]. Cohort studies also suggest that cognitive deficits correlate with reduced quality of life and limited ability to return to work, and delirium during hospitalization remains one of the most important modifiable risk factors for these impairments [8,28]. From an epidemiological and clinical perspective, neurological complications after ICU constitute a continuum: delirium affects approximately one third of patients and worsens short-term prognosis; ICU-AW occurs in a significant proportion of patients with severe, prolonged critical illness and complicates treatment; and long-term cognitive impairment constitutes a persistent health burden after discharge. These data justify the need for systematic prevention, early diagnosis and organized post-ICU care aimed at reducing long-term neurological sequelae [4,5,24,25,26,27,28].

## 6. Pathophysiology of Acute Brain Dysfunction in Critical Illness

The pathophysiology of neurological complications in intensive care is multifactorial and involves overlapping mechanisms related to the critical illness itself (e.g., sepsis, hypoxia, organ failure), the treatment (analgosedation, mechanical ventilation), and the ICU environment (sleep fragmentation, circadian rhythm disturbances). The common denominator of these processes is disruption of CNS homeostasis, leading to acute brain dysfunction and, in some patients, to persistent neurocognitive disorders after ICU discharge [29,30,31].

### 6.1. Neuroinflammation and Immune Dysregulation

One of the key mechanisms of delirium and encephalopathy in critical illness is neuroinflammation induced by a systemic inflammatory response. Current reviews emphasize the role of proinflammatory cytokines, microglial activation, and secondary neuronal dysfunction, clinically manifested by attention deficits, fluctuations in consciousness, and executive dysfunction. These processes are exacerbated in the course of sepsis and constitute the pathophysiological core of sepsis-associated encephalopathy, in which brain dysfunction develops without direct CNS infection and encompasses a spectrum ranging from delirium to coma [7,14,29,30,32].

### 6.2. Dysfunction of the Blood–Brain Barrier, Microcirculation, and Cerebral Metabolism

An important link in the neurological complications of ICU patients is increased permeability of the blood–brain barrier (BBB) and microcirculatory disorders, leading to impaired perfusion, oxidative stress, and neuronal metabolic dysfunction of neurons. In SAE and other critical illnesses, the interaction of BBB damage, endothelial dysfunction, and impaired autoregulation of cerebral blood flow is emphasized, which contributes to acute impairment of cognitive functions and consciousness [7,14,33]. Newer concepts also point to the possible involvement of impaired mechanisms of CNS “clearance” of toxic metabolites as a factor exacerbating brain dysfunction in sepsis [34].

### 6.3. Disorders of Neurotransmission and Neuronal Networks

Delirium is associated with disturbances in the main neurotransmitter systems (cholinergic, dopaminergic, GABAergic) and with disorganization of the neuronal networks responsible for attention and stimulus integration. These mechanisms are secondary to neuroinflammation, metabolic disturbances, sedative drug effects, and sleep deprivation, which explains the heterogeneity of delirium phenotypes and its fluctuating course [29,30].

### 6.4. Sleep and Selective Rhythm as a Modulator of Brain Dysfunction

Sleep disturbances and circadian rhythm dysregulation are common in the ICU and may exacerbate the neuroinflammatory cascade and susceptibility to delirium. A review by Daou et al. synthesizes the mechanisms linking sleep fragmentation to brain dysfunction, highlighting alterations in neuroendocrine, immunological, and neurotransmission systems [35]. The importance of these observations is reflected in the PADIS guidelines, which emphasize minimizing excessive sedation, improving sleep quality, and using nonpharmacological interventions [1,2].

### 6.5. Mechanism of Long-Term Cognitive Impairment

Persistent cognitive impairment after critical illness (PICS cognitive component) is viewed as a result of the overlap of acute brain dysfunction, chronic inflammatory, vascular, and metabolic changes, and environmental and iatrogenic factors such as deep sedation, immobilization, and disturbed sleep [28,31]. Reviews synthesizing current evidence point to the convergence of inflammatory processes, vascular barrier dysfunction, perfusion abnormalities, and oxidative stress-dependent mechanisms as a common pathway leading to cognitive deficits [31]. The frequency of these impairments in short- and long-term follow-up highlights the clinical importance of early understanding of the pathophysiology and of implementing preventive strategies as early as the ICU hospitalization stage [27].

### 6.6. Neuromonitoring in the ICU: Toward a Brain-Centered Approach

In recent years, neuromonitoring has become a key component of intensive care, enabling continuous, real-time assessment of brain function, perfusion, and metabolic status in critically ill patients. In addition to traditional clinical examination and periodic neurological assessment, modern neuromonitoring integrates both noninvasive and invasive methods, providing a dynamic and personalized assessment of acute brain dysfunction.

Multimodal neuromonitoring refers to the simultaneous use and integration of multiple techniques, including electroencephalography (EEG), intracranial pressure (ICP), brain tissue oxygenation (PbtO_2_), near-infrared spectroscopy (NIRS), transcranial Doppler (TCD), and cerebral microdialysis. These methods provide complementary information on brain electrical activity, perfusion, oxygenation, and metabolic status, enabling a more comprehensive understanding of the pathophysiology of the critically ill brain [36,37].

Continuous EEG monitoring has become increasingly important not only for detecting non-convulsive seizures and status epilepticus, but also for prognostication and assessment of cerebral dysfunction in a wide range of ICU populations. Importantly, EEG abnormalities may precede clinical deterioration, supporting its role as an early biomarker of brain injury [38].

Cerebral oxygenation monitoring, particularly using near-infrared spectroscopy (NIRS), has gained attention as a non-invasive bedside tool reflecting regional cerebral oxygen saturation (rSO_2_). Recent prospective data suggest that reduced cerebral oxygenation is associated with a higher incidence of delirium in critically ill patients, supporting the hypothesis that impaired cerebral oxygen delivery may contribute to acute brain dysfunction [39]. Similarly, invasive monitoring of brain tissue oxygen tension (PbtO_2_) allows early detection of cerebral hypoxia even when conventional parameters such as ICP and cerebral perfusion pressure remain within normal ranges [40].

Integrating multimodal data enables a shift from protocolized to personalized treatment strategies. By combining physiological signals, patient-specific thresholds for cerebral autoregulation can be identified, perfusion targets optimized, and secondary brain damage reduced. Despite its growing popularity, the clinical implementation of multimodal neuromonitoring remains heterogeneous, with limited standardization and a need for high-quality, outcome-oriented research [36].

From a clinical perspective, neuromonitoring may play a pivotal role in the prevention and management of delirium and other forms of acute brain dysfunction. Continuous assessment of cerebral function and oxygenation provides an opportunity to detect subclinical deterioration early and guide interventions, such as optimizing sedation, hemodynamics, and ventilation. This aligns with the concept of proactive, brain-oriented intensive care, in which neurological integrity is treated as a primary therapeutic target rather than a secondary outcome [41].

## 7. Risk Factors of Neurological Complications in the ICU

The risk of neurological complications in the ICU—including delirium and encephalopathy, ICU-acquired weakness (ICU-AW), and consequently, long-term cognitive impairment—results from the overlap of patient predispositions and triggers related to the critical illness and its treatment. Clinically, it is useful to distinguish between non-modifiable (predisposing) and modifiable (potentially reducible through intervention) factors. Pathophysiologically, most of these factors converge on common pathways, including neuroinflammation, dysfunction of the blood–brain barrier and microcirculation, disturbances in neurotransmission, and dysregulation of sleep and circadian rhythms. In ICU-AW, these include increased muscle catabolism, energy deficits, and neuromuscular dysfunction [2,42,43].

### 7.1. Non-Modifiable Factors

#### 7.1.1. Delirium and Encephalopathy

A systematic review by Zaal et al. identifies classic predisposing factors for delirium and encephalopathy, including advanced age, preexisting dementia, and hypertension. The circumstances of ICU admission (e.g., emergency surgery, trauma) and the overall severity of the clinical condition, as assessed by the APACHE II scale and others, are also important. These factors reduce cognitive reserve and increase the brain’s susceptibility to perfusion disorders, neuroinflammation, and dysregulated neurotransmission, consistent with current pathophysiological models of delirium [42,43].

#### 7.1.2. ICU-Acquired Weakness

Secondary reviews and meta-analyses by Fuentes-Aspe et al. consistently associated ICU-AW with non-modifiable risk factors, including older age, female gender, and multiple organ failure. These observations include susceptibility to increased muscle catabolism, impaired skeletal muscle microcirculation, and mitochondrial dysfunction during the course of the disease [44].

#### 7.1.3. Long-Term Cognitive Impairment After ICU

Review data indicate that the risk of long-term cognitive decline increases in patients with lower cognitive reserve, particularly in older age and with pre-existing cognitive impairment. However, the strongest predictor remains the duration of delirium in the acute phase of the illness [43,45,46].

### 7.2. Modifiable Factors

#### 7.2.1. Modifiable Factors Associated with Delirium and Encephalopathy

Delirium and encephalopathy are among the most common and burdensome neurological complications in critically ill patients, associated with prolonged mechanical ventilation and ICU stay, increased mortality, and long-term cognitive consequences within the PICS [4,5,43]. Unlike non-modifiable factors, a significant portion of the risk stems from elements amenable to clinical intervention.

Modifiable risk factors can be grouped into several domains: depth and type of sedation, use of centrally acting medications (particularly benzodiazepines), mechanical ventilation and metabolic disorders, multi-organ dysfunction, and sleep and circadian rhythm disturbances (Table 1). Despite clinical heterogeneity, these elements converge on common pathophysiological mechanisms, including neuroinflammation, blood–brain barrier dysfunction, cerebral microcirculation disorders, dysregulated neurotransmission, and sleep fragmentation.

One of the best-documented triggers of delirium is excessive sedation and iatrogenically induced coma, often associated with mechanical ventilation. A review by Zaal et al. found that these factors are among the strongest predictors of delirium in the ICU [42]. Mechanistically, deep sedation disrupts neural networks responsible for attention and consciousness, exacerbates dysregulation of the GABAergic and dopaminergic systems, and leads to secondary disturbances of the sleep–wake rhythm [29,30]. For this reason, the SCCM PADIS guidelines (2018) and their 2025 update clearly recommend light sedation strategies, provided the patient’s clinical condition allows it [1,2]. Among the pharmacological factors, exposure to benzodiazepines, especially continuous infusions, plays a particularly important role. Data from systematic reviews and observational studies indicate a significant association between their use and an increased risk of delirium, which is explained by increased neurotransmission disturbances, deterioration of sleep architecture, and secondary effects on neuroplasticity [42,47,48].

Respiratory and metabolic disorders, including hypoxemia, hypercapnia, metabolic acidosis, and electrolyte disturbances, also constitute a significant group of modifiable factors. These factors impair cerebral metabolism, exacerbate microcirculatory dysfunction, and increase blood–brain barrier permeability, thereby increasing susceptibility to acute brain dysfunction [7,30,42].

Sleep and circadian rhythm disturbances resulting from noise, artificial lighting, and nighttime procedures in the ICU are equally important and often underestimated. The review by Daou et al. showed a strong association between delirium and environmental factors through neuroendocrine, immunological, and neurotransmission mechanisms, which justifies the inclusion of environmental interventions in delirium prevention strategies [35], in line with the PADIS guidelines and the bundle-based approach (e.g., ABCDEF) [1,2,49].

#### 7.2.2. Modifiable Factors Associated with ICU-AW

The reviews by Fuentes-Aspe et al. and the umbrella review by Zhang et al. indicate that the impact of modifiable risk factors for ICU-AW is heterogeneous, but consistently, elements related to the intensity of treatment and the severity of critical illness, such as exposure to selected drugs, renal replacement therapy, or the use of vasopressors, appear [44,50]. The metabolic component is also clinically important: a 2025 meta-analysis in mechanically ventilated patients emphasizes the role of metabolic disorders, including hyperglycemia, in the pathogenesis of neuromuscular dysfunction, which correlates with the concept of muscle energy and microcirculation disorders in critical illness [51].

#### 7.2.3. Modifiable Factors of Long-Term Cognitive Impairment

The most consistent and clinically relevant modifiable risk factor for long-term cognitive dysfunction remains delirium, particularly its duration. Both the review by Sakusic et al. and subsequent evidence syntheses indicate that the association between delirium and persistent cognitive deficits is the strongest and most consistently documented, whereas other hospitalization factors show less clear evidence [46]. From a pathophysiological perspective, these observations support the hypothesis that delirium represents a clinical phenotype of acute brain dysfunction, and that the persistence of neuroinflammatory processes, blood–brain barrier dysfunction, and neural network disruption may initiate a cascade leading to chronic cognitive impairment [43,45].

## 8. Delirium in the Intensive Care Unit

### 8.1. Epidemiology and Clinical Phenotypes

Delirium is one of the most common neurological complications in adult ICU patients. Meta-analyses of studies using validated diagnostic tools indicate that its prevalence is approximately 30–35%, reaching as high as 30–80% in high-risk populations, such as mechanically ventilated patients or those with severe multi-organ failure [4,5,52]. Clinically, three main phenotypes are distinguished: hypoactive, hyperactive, and mixed. The most frequently observed hypoactive phenotype remains the most underdiagnosed, despite being associated with a poorer prognosis. The diversity of phenotypes reflects distinct mechanisms of acute brain dysfunction, including neuroinflammation, neurotransmitter disturbances, and circadian rhythm dysregulation [4,52].

### 8.2. Screening and Diagnosis (CAM-ICU, ICDSC)

According to the SCCM PADIS guidelines (2018) and their 2025 update, routine delirium screening should be performed in all adult ICU patients for whom consciousness can be assessed [1,2]. The most commonly used tool is the Confusion Assessment Method for the ICU (CAM-ICU), which enables the diagnosis of delirium regardless of a patient’s verbal communication. An alternative is the Intensive Care Delirium Screening Checklist (ICDSC), which allows for a time-spectrum assessment [5,53,54]. Both tools are characterized by good sensitivity and high specificity and are recommended for both clinical practice and research [1,2]. Increasing importance is also being attached to assessing delirium severity, including the CAM-ICU-7 scale, whose scores correlate with ICU length of stay, mortality, and the risk of long-term cognitive impairment [12].

### 8.3. Differential Diagnosis of Delirium in ICU Patients

The diagnosis of delirium requires the exclusion of other causes of acute brain dysfunction, including metabolic and toxic encephalopathies, septic encephalopathy, acute structural CNS pathology, and nonconvulsive status epilepticus (NCSE) [7,55]. In cases of persistent or unexplained disturbances of consciousness, it is recommended to extend the diagnostic process with EEG (especially if NCSE is suspected) and neuroimaging (CT/MRI) in the case of focal symptoms or an atypical course. Concomitant assessment of metabolic parameters, renal and liver function, and exposure to centrally acting drugs is also necessary [15,56].

### 8.4. Prevention Strategies (ABCDE/ABCDEF Bundle)

Delirium prevention relies on a multicomponent, non-pharmacological approach based on the ABCDE/ABCDEF bundle strategies (Table 2). These include optimal pain control, avoidance of oversedation, daily attempts at arousal and spontaneous breathing, early mobilization, systematic monitoring of delirium, and family involvement [1,49]. Systematic reviews and implementation studies have shown that the bundle-based approach is associated with reduced rates of delirium, shorter duration of mechanical ventilation and ICU stay, and improved functional outcomes after discharge [49,57].

### 8.5. Pharmacological and Non-Pharmacological Management

The cornerstone of delirium treatment remains the identification and correction of reversible triggers, such as infection, metabolic disturbances, excessive sedation, or sleep deprivation [1,2]. Available evidence does not support the routine use of antipsychotics for the prevention or treatment of delirium; they may only be considered short-term in severe hyperactive forms with a risk to patient or staff safety [1,58]. Non-pharmacological interventions, including reorientation, sleep optimization, early mobilization, and minimizing exposure to drugs with delirogenic potential, play a key role [59].

### 8.6. Prognostic Implications of ICU Delirium

Delirium is an independent predictor of increased mortality, longer ICU and hospital stays, and the development of long-term cognitive impairment after discharge [5,43]. The duration and severity of delirium are of prognostic importance and correlate with the risk of the cognitive component of PICS [43]. Therefore, delirium should be viewed not only as a transient disturbance in the acute phase of the illness, but as a key marker and potential mediator of long-term neurological sequelae following intensive care.

## 9. Encephalopathies in the Intensive Care Unit

Encephalopathy in the intensive care unit (ICU) refers to a generalized, usually reversible brain dysfunction manifesting as a spectrum of symptoms ranging from subtle disturbances of attention and executive function to profound disturbances of consciousness and coma. In critically ill patients, encephalopathy typically has a multifactorial etiology and results from the overlap of sepsis, hypoxia, and/or hypercapnia, metabolic disturbances, organ failure, and the effects of centrally acting drugs, which significantly complicates clear causal classification in clinical practice [60]. In the ICU environment, encephalopathies often clinically overlap with delirium and coma, and the boundaries between these phenotypes of acute brain dysfunction are fluid. Therefore, encephalopathy is increasingly being viewed not as a distinct entity but as a component of the spectrum of acute brain dysfunction in critical illness, requiring a parallel approach, including systematic screening for delirium and causal diagnosis of impaired consciousness [7,14,60].

### 9.1. Sepsis-Associated Encephalopathy as a Model of Critical Illness-Related Encephalopathy

Sepsis-associated encephalopathy (SAE) is the best-described and clinically most representative model of critical illness encephalopathy. It is defined as brain dysfunction developing during sepsis in the absence of direct CNS infection, a detectable structural lesion, or another dominant cause of encephalopathy [7].

SAE is a diagnosis of exclusion and is characterized by significant heterogeneity in clinical presentation, ranging from delirium and fluctuating attention deficits to profound encephalopathy and coma. Current reviews emphasize the lack of a single diagnostic biomarker and the need to interpret neurological symptoms within the strict clinical context and dynamic course of sepsis [7,13,14].

The pathophysiology of SAE involves interactions among neuroinflammation, blood–brain barrier dysfunction, cerebral microcirculation disorders, and metabolic and neurotransmission abnormalities, leading to global disorganization of neural networks responsible for attention, consciousness, and executive functions [7,14]. These mechanisms make SAE a clinical “bridge” between acute brain dysfunction in the ICU and long-term neurocognitive sequelae observed after discharge [31].

From a prognostic perspective, SAE is associated with prolonged ICU stay, increased mortality, and a higher risk of long-term neuropsychiatric sequelae, highlighting its importance as a key phenotype of critical illness encephalopathy [7,14].

### 9.2. Other Encephalopathies in the ICU—A Pragmatic Classification

In addition to SAE, a number of other encephalopathies are observed in ICUs, which often coexist and overlap pathophysiologically. In clinical practice, a simplified, pragmatic classification based on the dominant pathogenic mechanism (Table 3) is useful, enabling targeted diagnosis and correction of potentially reversible factors [60].

### 9.3. Diagnostic Approach to Encephalopathy in the ICU

The diagnosis of encephalopathy in critically ill patients should be iterative and multifaceted, with an emphasis on identifying reversible causes of acute brain dysfunction. Current reviews recommend a systematic approach, including: (1) assessment of sedation depth and exposure to centrally acting drugs, (2) targeted laboratory testing for metabolic abnormalities and organ failure, (3) diagnosis of sepsis and infection, (4) neuroimaging in the case of focal symptoms or an atypical course, and (5) electroencephalography, particularly if nonconvulsive status epilepticus is suspected [7,60].

In the context of SAE, simultaneous confirmation of sepsis and exclusion of other potential causes of encephalopathy is crucial. The lack of clear diagnostic criteria underscores the need to integrate clinical, laboratory, and neurophysiological data and to foster close interdisciplinary collaboration in the ICU [7,13].

## 10. Seizures and Non-Convulsive Status Epilepticus in the ICU

Seizures and non-convulsive status epilepticus (NCSE) are significant, often underestimated, causes of secondary brain dysfunction in critically ill patients. In the intensive care unit (ICU), their diagnosis is particularly difficult due to sedation, mechanical ventilation, and concomitant encephalopathy, which is why they most often manifest as persistent or fluctuating disturbances of consciousness without overt motor symptoms [69].

From a clinical perspective, it is crucial that a significant proportion of seizures in the ICU are non-convulsive seizures (NCS), and NCSE may be the only reversible cause of failure to awaken after reduced sedation or worsening encephalopathy despite correction of metabolic and hemodynamic disturbances [56,69]. Therefore, NCS/NCSE should be viewed as an integral component of the spectrum of acute brain dysfunction in critical illness, rather than solely as an independent neurological entity.

### 10.1. Epidemiology and Clinical Relevance

Observational data indicate that a significant percentage of ICU patients undergoing electroencephalography (EEG) due to unexplained disturbances of consciousness demonstrate seizure activity, including NCS and NCSE, particularly in high-risk groups such as patients with sepsis, anoxic brain injury, acute structural CNS pathology, and severe encephalopathy [69,70]. The American Clinical Neurophysiology Society (ACNS) consensus statement emphasizes that the absence of clinical signs of seizures does not exclude significant epileptic activity, which may contribute to secondary brain damage and worsen prognosis [70].

The prognostic significance of NCS/NCSE depends largely on the etiology of the critical illness and the time to diagnosis. Early detection and treatment of potentially reversible seizure activity are important elements of neuroprotective management in the ICU [69].

### 10.2. When to Suspect NCS/NCSE in ICU Patients

In clinical practice, NCS/NCSE should be suspected in patients with persistent or unexplained disturbances of consciousness, particularly in situations such as failure to adequately awaken after sedation reduction, sudden neurological deterioration without an identifiable metabolic or hemodynamic cause, post-convulsive status epilepticus, and the presence of severe encephalopathy, including sepsis-associated encephalopathy [56,69].

**Figure 1:**
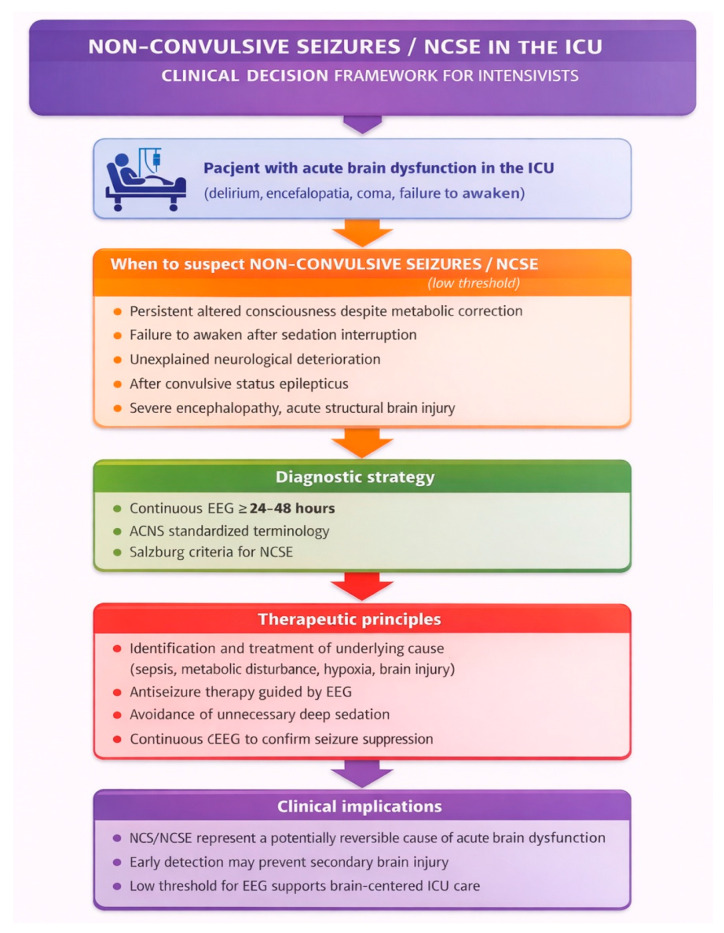
Non-convulsive seizures and non-convulsive status epilepticus in the intensive care unit: a pragmatic clinical decision framework.

In these contexts, electroencephalography—preferably in the form of continuous monitoring (cEEG)—is a key diagnostic tool, enabling the identification of “hidden” seizure activity, inaccessible to clinical assessment [70,71]. The procedure for the introduction is shown in Figure 1.

### 10.3. Diagnostic Criteria and Management Principles

The diagnosis of NCSE in the ICU is based on electroencephalographic criteria interpreted within a rigorous clinical context. The Salzburg Consensus Criteria (SCNC), published and validated in 2015 and subsequently confirmed in subsequent studies, are one of the most well-established diagnostic tools, reducing the risk of overdiagnosis of NCSE in patients with severe encephalopathy and nonspecific EEG patterns [56,72]. Their use in conjunction with ACNS terminology increases diagnostic consistency and supports rational therapeutic decisions [15].

Therapeutic management of NCS/NCSE in the ICU includes concurrent treatment of the underlying cause and antiepileptic therapy, with efficacy assessed by EEG, particularly in cases without clear clinical correlation. Continued cEEG monitoring is crucial to confirm resolution of seizure activity and detect recurrences, especially in patients with profound encephalopathy and during sedation [56,69,70,71].

### 10.4. Clinical Significance

From a clinical perspective, NCS and NCSE should be considered potentially reversible components of acute brain dysfunction in the ICU, requiring high diagnostic vigilance and a low threshold for EEG implementation in patients with impaired consciousness. This approach aligns with the contemporary concept of brain-centered intensive care, which emphasizes brain protection as an integral element of critical illness treatment [69,70].

## 11. Cerebrovascular Injury as a Component of Acute Brain Dysfunction in Critical Illness

Cerebrovascular complications in patients treated in the intensive care unit encompass a heterogeneous group of acute and subacute brain injuries, which are increasingly being viewed not as isolated vascular events but as a phenotype of brain damage associated with critical illness. Unlike classic stroke syndromes, cerebrovascular injury in the ICU often develops based on generalized endothelial dysfunction, microcirculatory disturbances, inflammatory coagulopathy, and hemodynamic and blood gas instability, leading to subclinical or minimally symptomatic brain damage with potentially significant cognitive consequences [73,74,75,76]. In this context, cerebrovascular complications should be considered an integral component of acute brain dysfunction in critical illness, closely related to delirium, encephalopathy, and long-term neurocognitive consequences after ICU discharge.

### 11.1. Microvascular and Endothelial Mechanisms of Cerebrovascular Injury

The central mechanism of cerebrovascular injury in critical illness is endothelial and cerebral microcirculation dysfunction, induced by a generalized inflammatory response, activation of the coagulation system, and impaired autoregulation of cerebral blood flow. Data from reviews of sepsis-induced coagulopathy (SIC) and DIC indicate a close coupling of inflammatory processes with coagulation activation and secondary fibrinolysis inhibition, which simultaneously promote microthrombosis and subsequent bleeding complications [75,76].

In sepsis, severe respiratory failure, and during ECMO therapy, increased damage to the blood–brain barrier, perfusion disturbances, and increased susceptibility to cerebral microbleeds are also observed, creating a pathophysiological bridge between critical illness encephalopathy and CNS vascular damage [74,77,78].

### 11.2. Critical Illness—Associated Cerebral Microbleeds

A specific phenotype of cerebrovascular injury in the ICU is critical illness–associated cerebral microbleeds (CMBs), described particularly in patients with severe ARDS, sepsis, long-term mechanical ventilation, and after ECMO treatment. Systematic and scoping reviews indicate that microbleeds are typically located in the white matter, corpus callosum, and subcortical structures, distinguishing this pattern from hypertensive or amyloid microangiopathy [74,79]. The pathogenesis of CMBs in critical illness is associated with a combination of hypoxemia, endothelial dysfunction, hemostasis disturbances, and rapid perfusion changes. Their presence correlates with disease severity, poorer functional prognosis, and increased mortality [80].

From a clinical point of view, CMBs are a marker of diffuse brain damage that may coexist with delirium and encephalopathy and sometimes explains persistent cognitive deficits after discharge from the ICU.

### 11.3. Cerebrovascular Injury in High-Risk ICU Settings

The risk of cerebrovascular injury increases significantly in specific clinical settings within the ICU. Patients with sepsis have an increased risk of stroke, persisting for months after the septic episode, suggesting a long-term prothrombotic phenotype and chronic endothelial dysfunction [77,78]. Similar observations have been made in patients with severe COVID-19, in whom strokes and microbleeds have been associated with increased inflammation and coagulopathy [81,82].

Patients treated with ECMO constitute a particularly high-risk group. Meta-analyses and large cohorts have demonstrated a significant incidence of intracranial hemorrhage, often with minimal symptoms, the pathogenesis of which is associated with the need for anticoagulation, impaired hemostasis, and rapid changes in blood gases and perfusion during the peri-cannulation period [83,84].

### 11.4. Diagnostic and Clinical Implications in the ICU

Diagnosing cerebrovascular injury in the ICU is significantly hampered by sedation and limited neurological examination options. Therefore, an approach based on high clinical vigilance, regular neurological assessment during sedation reduction, and a low threshold for neuroimaging (CT/MRI) in cases of unexplained disturbances of consciousness, focal symptoms, or inadequate awakening is recommended [85,86,87].

In clinical practice, cerebrovascular injury should be viewed as part of the spectrum of acute brain dysfunction, which can exacerbate delirium, complicate the course of critical illness encephalopathy, and contribute to long-term cognitive impairment after discharge. This approach aligns with the concept of brain-centered intensive care, which emphasizes the need for integrated vascular, metabolic, and neurocognitive assessment in critically ill patients [73,74].

## 12. Peripheral Nervous System Complications as a Component of Post-Intensive Care Syndrome

Peripheral nervous system (PNS) complications in patients treated in the intensive care unit (ICU) constitute a significant component of the spectrum of critical illness outcomes and are currently viewed primarily in the context of Post-Intensive Care Syndrome (PICS). The most common and clinically significant phenotype of these complications is ICU-acquired weakness (ICU-AW), which includes critical illness polyneuropathy (CIP), critical illness myopathy (CIM), and the mixed form (CIPNM) [10,25].

Unlike acute central nervous system complications, ICU-AW rarely manifests as an isolated event. It most often develops while prolonged critical illness, coexisting with delirium, encephalopathy, and multiorgan dysfunction. Its clinical significance stems from the long-term functional consequences and impact on quality of life after ICU discharge [25,88].

### 12.1. ICU-Acquired Weakness as a Dominant PNS Phenotype in Critical Illness

ICU-acquired weakness is defined as generalized, symmetrical muscle weakness that develops during critical illness and is unexplained by other neurological causes, with frequent involvement of proximal and respiratory muscles [25,88]. Systematic reviews and meta-analyses indicate that the incidence of ICU-AW reaches 40–50%, with a higher incidence in populations with sepsis, prolonged mechanical ventilation, and multi-organ failure [24,25].

The pathophysiology of ICU-AW is multifactorial and includes impaired neuromuscular excitability, mitochondrial dysfunction, increased muscle catabolism, microcirculatory disorders, and the effects of immobilization and therapeutic interventions in the ICU [25,89]. These mechanisms partially overlap with the pathophysiology of acute brain dysfunction, supporting the concept of a common “systemic” basis for the neurological sequelae of critical illness. From a clinical perspective, ICU-AW significantly prolongs mechanical ventilation duration, increases the risk of extubation failure, and leads to long-term limitations in physical function, making it a key determinant of the physical component of PICS [25,26].

### 12.2. Diagnostic Approach to ICU-Acquired Weakness in the ICU

The diagnosis of ICU-AW requires first excluding central nervous system pathology and acute vascular events, which may mask or mimic peripheral muscle weakness. In cooperative patients, the primary screening tool remains a standardized assessment of muscle strength using the Medical Research Council sum score (MRCss), while handgrip dynamometry may be a useful tool for early identification of patients at risk of ICU-AW [10,25].

In uncooperative patients or to differentiate between phenotypes (CIP vs. CIM), electrophysiological studies, including electromyography and nerve conduction studies, play a key role. Accumulating evidence also indicates the increasing usefulness of ultrasound methods in assessing muscle mass and architecture, which can support monitoring of the course of ICU-AW over time [90,91,92].

### 12.3. Focal Peripheral Nerve Injuries in Critically Ill Patients

In addition to generalized ICU-AW, focal peripheral nerve and plexus injuries are observed in the ICU, resulting from prolonged immobilization, compression, and improper positioning of patients, particularly during mechanical ventilation in the prone position. Recent reports, particularly from patients with severe ARDS, indicate a significant incidence of brachial plexus injuries and mononeuropathy of the upper limbs [93,94].

These complications, although often considered “iatrogenic,” have significant functional significance and can further delay post-ICU rehabilitation. Recent reviews emphasize the importance of standardized positioning protocols, pressure point protection, and early identification of neurological symptoms to reduce the risk of permanent deficits [93,95,96,97].

### 12.4. ICU-Acquired Weakness and Its Relationship with Delirium and PICS

Accumulating evidence indicates that ICU-AW rarely occurs in isolation and often coexists with delirium and critical illness encephalopathy. These observations suggest shared pathophysiological mechanisms, including a systemic inflammatory response, microcirculatory dysfunction, and energy imbalances that simultaneously impact the central and peripheral nervous systems [25,43].

From a long-term perspective, ICU-AW constitutes a key physical component of PICS and significantly correlates with limited independence, reduced quality of life, and reduced ability to return to work after ICU discharge. In this context, peripheral nervous system complications should be considered not as a separate neurological problem but as an integral component of the multidimensional consequences of critical illness, requiring a coordinated approach encompassing prevention, early rehabilitation, and post-ICU care [26,98].

## 13. Long-Term Cognitive Dysfunction After ICU Discharge

Long-term cognitive dysfunction after intensive care is a key component of post-intensive care syndrome (PICS) and encompasses persistent, clinically significant deficits in cognitive functions such as attention, memory, executive function, and processing speed that emerge after an episode of critical illness and persist for months or years after discharge from the intensive care unit (ICU). These impairments often coexist with psychiatric symptoms, including depression, anxiety, and post-traumatic stress disorder, as well as physical limitations, leading to significant functional, social, and occupational burden for ICU survivors [8,28,98].

Current data indicate that long-term cognitive sequelae are not solely the consequence of a single factor, but are the result of the cumulative impact of acute brain dysfunction (particularly delirium and sepsis-associated encephalopathy), a generalized inflammatory response, microcirculatory disturbances, and iatrogenic factors, which is consistent with the concept of critical illness as an event with lasting neurological implications.

### 13.1. Prevalence and Temporal Trajectory of Cognitive Impairment After ICU

The incidence of cognitive impairment after ICU discharge varies significantly and depends on the severity of the critical illness, the characteristics of the study population, the diagnostic tools used, and the timing of the post-discharge assessment. Systematic reviews and cohort studies demonstrate that cognitive deficits can persist for many months or even years, with significant heterogeneity over time—from gradual improvement to persistent or progressive cognitive impairment [45,99,100].

The most recent quantitative synthesis of data from 2015–2025 is the proportional observational meta-analysis by Ho et al. (2025), which demonstrated a high incidence of post-intensive care cognitive impairment in both short- and long-term follow-up, while also highlighting significant differences stemming from study methodology and assessment tools used [27]. These data indicate an urgent need to standardize cognitive function measurement in PICS studies.

### 13.2. Cognitive Phenotype and Affected Domains

Neuropsychological analyses of individuals discharged from the ICU consistently indicate that executive functions, information processing speed, attention, and working memory are most frequently impaired. This profile of deficits significantly impacts the ability to function independently, return to work, and quality of life after critical illness [45,101].

Prospective cohorts of patients who experienced delirium during their ICU stay showed significantly poorer performance on tests assessing information processing speed (Trail Making Test A) and executive functions (Trail Making Test B) in the post-discharge period. At the same time, tools such as the Repeatable Battery for the Assessment of Neuropsychological Status (RBANS) and the TMT have been shown to be more sensitive in detecting cognitive deficits after ICU than the commonly used Mini-Mental State Examination, which may not identify subtle but clinically significant impairments [101].

### 13.3. Risk Factors and Pathophysiological Links with Acute Brain Dysfunction

Risk factors for long-term cognitive dysfunction include both premorbid factors, such as age and previous cognitive function, and factors related to the course of critical illness. Delirium episodes, their duration, and severity play a particularly important role, as repeatedly confirmed in cohort studies and systematic reviews [45,99].

Additional potentially modifiable risk factors include exposure to benzodiazepines and other centrally acting medications, deep and prolonged sedation, hypoxemia, and metabolic disturbances. These data support the hypothesis that long-term cognitive dysfunction is an extension of acute brain dysfunction developing during critical illness, rather than a distinct, late complication [45,99,100].

### 13.4. Clinical Implications and Integration Within the PICS Framework

From a clinical perspective, long-term cognitive dysfunction after ICU is one of the most significant determinants of quality of life for individuals after critical illness. Its presence is associated with limited independence, difficulties returning to work, and increased demand for long-term care, which has significant implications for both patients and healthcare systems [28,98].

Current recommendations emphasize the need to treat cognitive impairment as an integral component of PICS, requiring early identification, monitoring, and coordinated management after ICU discharge. This approach pathophysiologically and clinically integrates all stages described in this review—from acute brain dysfunction in the ICU, through its phenotypes (delirium, encephalopathy, seizures), to long-term neurocognitive sequelae in patients after intensive care [26,98].

## 14. Main Challenges in the Treatment of Neurological Complications in ICU Patients and Strategies to Reduce Them

Neurological complications in patients treated in the intensive care unit, including disturbances of consciousness (delirium and encephalopathies), seizures and nonconvulsive status epilepticus, cerebrovascular complications, ICU-acquired weakness, sleep disturbances, and the cognitive and functional components of post-intensive care syndrome, are characterized by a complex, multifactorial pathogenesis. Their diagnosis is often delayed or incomplete due to the need for sedation and mechanical ventilation, limited opportunities for conducting a reliable neurological examination, and the overlap of metabolic disorders and iatrogenic factors.

Current clinical guidelines and systematic reviews clearly indicate that effective reduction in neurological burden in this population requires implementing a bundled approach that integrates strategies for analgesia and sedation, prevention and early detection of delirium, early mobilization, and interventions to improve sleep quality. Acute care should be complemented by a planned, multidisciplinary rehabilitation pathway and structured post-ICU discharge follow-up, focused on early identification and treatment of neurological sequelae and PICS components. Table 4 presents key clinical challenges and strategies for reducing neurological sequelae in patients hospitalized in the intensive care unit.

## 15. Knowledge Gaps and Future Research Directions

Despite significant progress in recognizing and reducing “acute brain dysfunction” in the ICU, the current literature from 2015–2025 indicates persistent gaps: heterogeneity in definitions and outcome measures, limited comparability across studies, insufficient validation of biomarkers, and a lack of standardization of long-term cognitive and functional endpoints [9,52,102,110].

Table 5 summarizes the significant knowledge gaps and proposes directions for future research.

## 16. Discussion

This narrative review presents the neurological complications of critical illness as a spectrum of interrelated clinical phenotypes rather than a collection of distinct disease entities. The data indicate that acute brain dysfunction developing during the intensive care unit (ICU) stay—manifested by delirium, sepsis-associated encephalopathy, nonconvulsive seizures, or vascular injury—is the starting point for long-term neurological sequelae, including persistent cognitive dysfunction and components of post-intensive care syndrome (PICS) [8,25,28,43,98].

Delirium remains the best-characterized and most documented clinical marker of acute brain dysfunction in the ICU, and its frequency, duration, and severity are consistently associated with adverse neurological and cognitive outcomes after discharge [8,45]. However, increasing evidence indicates that delirium should not be considered an isolated clinical entity but rather as part of a broader continuum of critical illness encephalopathy, which also includes subclinical disturbances of brain bioelectrical activity and episodes of nonconvulsive seizures [43,100].

In this context, sepsis-associated encephalopathy (SAE) represents a model of generalized brain dysfunction induced by an inflammatory response, microcirculatory disturbances, and blood–brain barrier dysfunction, and constitutes a common pathophysiological basis for many observed neurological phenotypes [25]. Similarly, the increasing availability of continuous EEG monitoring reveals a significant frequency of nonconvulsive seizures and nonconvulsive status epilepticus in patients with unexplained disturbances of consciousness, which emphasizes the importance of latent epileptic activity as a potential factor worsening neurological prognosis [100]. A significant element of this spectrum is also vascular complications of the central nervous system, which—although relatively less frequently recognized clinically—may coexist with encephalopathy and delirium, further exacerbating long-term cognitive deficits [28]. In contrast, ICU-acquired weakness, encompassing critical illness polyneuropathy and myopathy, highlights the systemic nature of critical illness and its simultaneous impact on the central and peripheral nervous systems, reinforcing the concept of PICS as a multidimensional syndrome [25,26,98].

From a clinical perspective, the presented data support the need to move away from a fragmented approach to neurological complications in the ICU toward an integrated model in which early identification of acute brain dysfunction, its monitoring, and prevention of iatrogenic factors are key elements of the strategy to improve long-term neurological and functional outcomes in patients after critical illness [26,98].

## 17. Conclusions

The neurological complications of critical illness constitute a coherent clinical and pathophysiological spectrum, the central element of which is acute brain dysfunction that develops during intensive care unit treatment. Delirium, encephalopathy, nonconvulsive seizures, vascular injury, and ICU-acquired weakness are not distinct, independent phenomena but rather coexisting phenotypes of the body’s systemic response to severe illness and intensive care.

Long-term cognitive impairment after ICU discharge should be viewed as a continuation of processes initiated in the acute phase, rather than as a late, isolated complication. An integrated, brain-centered approach to ICU care—including brain function monitoring, delirium prevention, rational sedation, and early rehabilitation—is key to limiting the neurological consequences of critical illness and improving patients’ quality of life after intensive care.

## Figures and Tables

**Table 1:** Modifiable risk factors for acute brain dysfunction in the ICU.

Domain	Examples	Pathophysiological Mechanism
Sedation	Deep sedation, benzodiazepines	Neurotransmission imbalance, network suppression
Respiratory	Hypoxemia, hypercapnia	Impaired cerebral metabolism
Metabolic	Electrolyte disorders, acidosis	Neuronal dysfunction
Environmental	Noise, light, sleep disruption	Circadian dysregulation
Hemodynamic	Hypotension, perfusion variability	Microcirculatory dysfunction

**Table 2:** Bundle-based strategies for delirium prevention in ICU patients.

Component	Intervention	Clinical Effect
A	Pain control	Reduced stress response
B	Spontaneous awakening/breathing trials	Reduced sedation exposure
C	Sedation optimization	Lower delirium risk
D	Delirium monitoring	Early detection
E	Early mobilization	Reduced ICU-AW
F	Family engagement	Psychological stabilization

**Table 3:** Pragmatic classification of encephalopathies in the intensive care unit [7,14,61,62,63,64,65,66,67,68].

Dominant Mechanism	Clinical Examples	Key Diagnostic Features
Inflammatory and septic encephalopathies	Sepsis-associated encephalopathy (SAE)	Diagnosis of exclusion; association with sepsis; no CNS infection
Metabolic and toxic encephalopathies	Toxic-metabolic encephalopathy, hypoglycemia, dysnatremia	Fluctuating pattern; correlation with metabolic disorders
Organ failure encephalopathies	Hepatic, uremic encephalopathy	Depends on the severity of organ failure; improvement after causal treatment
Hypoxic-perfusion encephalopathies	Hypoxic encephalopathy, cerebral autoregulation disorders	Relationship to hypoxia, hypotension, perfusion disorders
Mixed encephalopathies	Combination of the above	The most common phenotype in the ICU—overlapping mechanisms

**Table 4:** Key clinical challenges and strategies to reduce neurological sequelae of ICU [1,2,15,25,28,44,49,70,98,102,103,104,105,106,107,108,109].

Area/Challenge in the ICU	Mechanism/Consequence	Strategy in the ICU	Post-ICU Strategy
Underestimation of delirium and “acute brain dysfunction”	Delirium may be masked by sedation; lack of systematic screening → delayed interventions; delirium is associated with a poorer prognosis and may correlate with later PICS deficits	Implementation of PADIS standards (systematic pain assessment, optimization of sedation/analgesia, avoidance of oversedation, routine assessment of delirium), ABCDEF practices	Structured post-ICU follow-up (cognitive/mental/functional assessment), patient and caregiver education, referral to neuropsychologist for symptomatic deficits; post-ICU clinic models as a coordination platform
Sedation/analgesia as a risk factor for neurological complications	Excessive or suboptimal sedation complicates neurological assessment and may increase the risk of delirium and delay mobilization.	Implementation of PADIS recommendations	Continuation of “delirium-informed care” in medical units: avoiding drugs with a high deliriogenic potential, working on sleep, monitoring anxiety and depression disorders within the PICS framework
Limited access to EEG/cEEG → NCS/NCSE omission	Non-convulsive seizures/NCSE often manifest only with disturbances of consciousness; the lack of EEG/cEEG makes diagnosis and assessment of treatment effectiveness difficult	Low threshold for EEG/cEEG, following the ACNS consensus—standardization of description; monitoring algorithms in patients with unexplained encephalopathy	Neurological follow-up plan, seizure education, and outpatient antiepileptic drug therapy optimization
ICU-acquired weakness (CIP/CIM) and delayed rehabilitation	ICU-AW increases the difficulty of weaning from the ventilator and is associated with poorer short- and long-term outcomes	Early mobilization and bedside rehabilitation, minimizing sedation, managing risk factors (e.g., hyperglycemia); standardized strength assessment (MRC sum score) and electrophysiological diagnostics as needed	Post-ICU rehabilitation programs (physiotherapy, performance training, occupational therapy) and function monitoring (mobility); in the case of persistent deficits – multidisciplinary rehabilitation using the PICS model
Sleep and circadian rhythm disorders	Noise/light/nighttime interventions exacerbate sleep fragmentation; sleep disturbances are associated with delirium and poorer well-being after discharge	Environmental interventions (noise/light reduction, stopwatches, alarm optimization), care planning—sleep improvement in patients ≥ 65 years in the ICU indicates the possibility of reducing the burden of delirium in a multi-component intervention model	Continued sleep hygiene, identification and treatment of insomnia/parasomnias; psychological support, especially for comorbid anxiety/PTSD within PICS/PICS-F
Fragmentation of care after discharge (lack of continuity of neurological treatment)	PICS encompasses cognitive, mental, and physical domains; without a follow-up plan, deficits persist, caregiver burden increases, and the risk of rehospitalization increases	Identify patients at high risk for PICS (delirium, prolonged ventilation, sepsis, ICU-AW) and plan discharge pathway	Post-ICU clinics, rehabilitation coordination, cognitive screening tests
Family Burden (PICS-F) and Clinical Communication	Caregiver stress affects the patient’s recovery process and cooperation; psychological and socioeconomic problems in the family are increasing	Family engagement as part of ABCDEF; communication and family participation in care	Support and education programs, caregiver burden assessment; intervention components in post-ICU clinics include PICS-F
Uncertain efficacy of single interventions after ICU	Some interventions, e.g., telehealth or complex programs, have mixed results; different study populations, measures, and implementations.	Preferring multi-component interventions in the ICU (ABCDEF/PADIS) and realistic resource planning	Critical implementation of post-ICU care programs (telemedicine/care coordinator) with evaluation of their outcomes

ICU—Intensive Care Unit; ICU-AW—Intensive Care Unit–Acquired Weakness; CIP—Critical Illness Polyneuropathy; CIM—Critical Illness Myopathy; NCSE—Non-Convulsive Status Epilepticus; NCS—Non-Convulsive Seizures; EEG—Electroencephalography; cEEG—Continuous Electroencephalography; PICS—Post-Intensive Care Syndrome.

**Table 5:** Research gaps and directions for future research.

Area	Knowledge Gaps	Methodological Limitations of the Research	Future Research Directions/Strategies
**Methodological limitations of existing studies**	Lack of consistent definitions of brain dysfunction in critical illness; limited generalizability of findings across ICUs	Patient selection, lack of post-discharge follow-up, differences in sedation and ICU protocols, insufficient baseline cognition	Multicenter studies with predefined protocols, better characterization of pre-ICU cognitive status, harmonization of exposure reporting (sedation, delirium, hypoxia)
**Need for standardized cognitive outcomes**	The high variability of cognitive assessment tools after ICU makes meta-analyses and translation into practice difficult.	Use of tests with low sensitivity to PICS domains, lack of uniform standards and reporting	Develop and implement standardized core outcome sets in ICU research and implement consensus recommendations for PICS assessment, favoring measures encompassing executive domains and processing speed
**Emerging biomarkers and neuroimaging**	Lack of biomarkers with sufficient reproducibility and predictive value for routine use; unclear translational pathway to clinical decisions	Small samples, different sampling time points, diverse definitions of delirium/encephalopathy, in neuroimaging – limited availability and heterogeneous protocols	Multimodal and longitudinal studies: biomarker panels (e.g., markers of neuronal/astroglial damage), clinical phenotypes, EEG/neuroimaging; validation in independent cohorts; development of mechanistic endotyping
**Digital and personalized approaches in ICU neurology**	Limited number of high-quality RCTs for digital interventions affecting delirium/PICS; unclear which components are the “active ingredient”	Heterogeneous interventions (apps, telecare, family tools), different endpoints and duration; implementation challenges and user acceptance	Designing human-centered interventions, with process evaluation and selection of standardized outcomes; personalization according to risk (e.g., delirium) and patient/family resources; developing digitally supported post-ICU care

ICU—Intensive Care Unit; ICUs—Intensive Care Units; PICS—Post-Intensive Care Syndrome; RCTs—Randomized Controlled Trials; EEG—Electroencephalography.

## Data Availability

At the correspondence author.

## Abbreviations

ABCDEF bundle	Assess, prevent, and manage pain; Both spontaneous awakening and breathing trials; Choice of analgesia and sedation; Delirium assessment, prevention, and management; Early mobility and exercise; Family engagement
ABCDE bundle	Awakening and Breathing coordination; Choice of sedation; Delirium monitoring and management; Early mobility
ACNS	American Clinical Neurophysiology Society
ARDS	Acute respiratory distress syndrome
BBB	Blood–brain barrier
CAM-ICU	Confusion Assessment Method for the Intensive Care Unit
CAM-ICU-7	Confusion Assessment Method for the Intensive Care Unit–7 severity scale
CIM	Critical illness myopathy
CIP	Critical illness polyneuropathy
CIPNM	Critical illness polyneuropathy and myopathy
CMBs	Cerebral microbleeds
cEEG	Continuous electroencephalography
CI	Confidence interval
COVID-19	Coronavirus disease 2019
DIC	Disseminated intravascular coagulation
ECMO	Extracorporeal membrane oxygenation
EEG	Electroencephalography
GABA	Gamma-aminobutyric acid
ICDSC	Intensive Care Delirium Screening Checklist
ICU	Intensive care unit
ICU-AW	Intensive care unit–acquired weakness
MeSH	Medical Subject Headings
MMSE	Mini-Mental State Examination
MR	Magnetic resonance imaging
MRCss	Medical Research Council sum score
NCSE	Non-convulsive status epilepticus
NCS	Non-convulsive seizures
ICU	Intensive Care Unit)
CNS	Central nervous system
PADIS	Pain, Agitation/Sedation, Delirium, Immobility, and Sleep Disruption guidelines
PICS	Post-Intensive Care Syndrome
PNS	Peripheral nervous system
RBANS	Repeatable Battery for the Assessment of Neuropsychological Status
RR	Relative risk
SAE	Sepsis-associated encephalopathy
SCNC	Salzburg Consensus Criteria
SCCM	Society of Critical Care Medicine
SIC	Sepsis-induced coagulopathy
TK	Computed tomography
TMT	Trail Making Test
